# Elevated levels of eEF1A2 protein expression in triple negative breast cancer relate with poor prognosis

**DOI:** 10.1371/journal.pone.0218030

**Published:** 2019-06-20

**Authors:** Fabiola Giudici, Elisabetta Petracci, Oriana Nanni, Cristina Bottin, Maurizio Pinamonti, Fabrizio Zanconati, Bruna Scaggiante

**Affiliations:** 1 Biostatistics Unit, Department of Medical, Surgical and Health Sciences, University of Trieste, Cattinara Academic Hospital, Trieste, Italy; 2 Unit of Biostatistics and Clinical Trials, Istituto Scientifico Romagnolo per lo Studio e la Cura dei Tumori, Meldola, Italy; 3 Department of Medical, Surgical and Health Sciences, University of Trieste, Cattinara Academic Hospital, Trieste, Italy; 4 Department of Life Sciences, University of Trieste, Trieste, Italy; University of South Alabama Mitchell Cancer Institute, UNITED STATES

## Abstract

Eukaryotic elongation factor 1 alpha 2 (eEF1A2) is a translation factor selectively expressed by heart, skeletal muscle, nervous system and some specialized cells. Its ectopic expression relates with tumorigenesis in several types of human cancer. No data are available about the role of eEF1A2 in Triple Negative Breast Cancers (TNBC). This study investigated the relation between eEF1A2 protein levels and the prognosis of TNBC. A total of 84 TNBC diagnosed in the period 2002–2011 were included in the study. eEF1A2 protein level was measured in formalin-fixed paraffin-embedded tissues by immunohistochemistry in a semi-quantitative manner (sum of the percentage of positive cells x staining intensity) on a scale from 0 to 300. Cox regression assessed the association between eEF1A2 levels and disease-free survival (DFS) and breast cancer-specific survival (BCSS). Elevated values of eEF1A2 were associated with older age at diagnosis (p = 0.003), and androgen receptors positivity (p = 0.002). At univariate Cox analysis, eEF1A2 levels were not significantly associated with DFS and BCSS (p = 0.11 and p = 0.08, respectively) whereas adjusting for stage of disease, elevated levels of eEF1A2 protein resulted associated with poor prognosis (HR = 1.05, 95% CI: 1.01–1.11, p = 0.04 and HR = 1.07, 95% CI: 1.01–1.14, p = 0.03 for DFS and BCSS, respectively). This trend was confirmed analyzing negative versus positive samples by using categorized scores. Our data showed a negative prognostic role of eEF1A2 protein in TNBC, sustaining further investigations to confirm this result by wider and independent cohorts of patients.

## Introduction

Breast cancer is the most common cancer in women, and the second most frequent cause of cancer-related deaths in women worldwide [1,2]. Prognostic factors include histological features (histological type, histological grade, lymphovascular invasion), tumor size, lymph node status, steroid hormone receptors status and age [3–5]. Molecular stratification based on gene expression profiling revealed that breast cancers could be classified in the so-called intrinsic subtypes-Luminal A, Luminal B, HER2-enriched, basal-like, and normal-like- [6], which correlate with the efficacy of chemotherapy and life expectancy [7].The St. Gallen International Expert Consensus proposed a method for classifying breast cancer into 4 subtypes using immunostaining: Luminal A, Luminal B, Human Epidermal growth factor Receptor 2 (HER2) positive and triple negative- type breast cancer (TNBC) [8]. Approximately 15–20% of all breast cancers are triple-negative breast cancer (TNBC) due to the lack of expression of three proteins: estrogen receptor (ER), progesterone receptor (PR), and HER2 [9].

TNBC tends to be more aggressive than other breast cancer subtypes, with a higher prevalence in African-Americans and more frequently affecting younger patients (average age <50 years) [1,2,10]. The clinical-pathological parameters consist of large tumors size, multiple apoptotic cells, high proliferative index, poor differentiation, central necrosis. The major histological type of TNBC is not otherwise specified (NOS) ductal and less commonly medullary [11]. TNBC is highly malignant, prone to metastasis and relapse, and, therefore, it has a poorer prognosis and a greater risk of mortality than other subtypes [12].

Currently, breast cancer’s therapeutic options are highly dependent on targeting ER, PR, or HER2. Unfortunately, TNBC cannot benefit from endocrine or targeted therapies [13,14]. Although TNBC patients can initially respond to the combination of chemotherapy, treatment failure and disease recurrence continue to be a clinical challenge [15]. The Food and Drug Administration (FDA)-approved biomarkers can be surrogate, prognostic, predictive, or pharmacodynamics [16] and some of them provided a cornerstone of modern cancer therapies, as the case of prognostic and predictive biomarkers p53 [17] and Ki-67 [18] that gained clinical utility in breast cancer too [19]. Unfortunately, these FDA biomarkers have not demonstrated significant clinical utility in the management of TNBC [20]. The research on cancer-related molecules in TNBC has grown considerably in recent years and some targeted agents are currently studying as candidates for therapy [21]. However, the possibility to anticipate the response to therapies and the risk of recurrence in TNBC by using tissue or blood biomarkers remains an important goal which has not been reached yet.

Among cancer-related biomarkers, eukaryotic Elongation Factor 1 Alpha (eEF1A) proteins have gained interest as potential predictors of cancer onset and progression in many human solid and hematopoietic cancers. Two main isoforms of eEF1A, namely eEF1A1 and eEF1A2, were identified and they play an important role in the elongation of nascent polypeptides during the protein synthesis by binding the aminoacylated tRNAs and carrying them to ribosomes. In particular, the eEF1A2 was first recognized as a tissue-specific variant of eEF1A1 (formerly known as EF-1α) in the early 1990s [22,23]. The isoforms are encoded by separate loci, EEF1A1 in 6q13 and EEF1A2 in 20q11, but the resulting proteins are highly homologous sharing 92% identity and 98% similarity. It is worth noting that their expression pattern is markedly different: eEF1A1 is expressed ubiquitously, whereas eEF1A2 expression after birth is limited to the heart, skeletal muscle, brain and to some specialized cells [23–25].

The eEF1A proteins have many translation-independent roles, as they are involved in various important cellular mechanisms: embryogenesis, senescence, cell proliferation, apoptosis, cytoskeletal organization and protein degradation. eEF1A1 is a major cytoskeletal remodeling factor by binding actin, it displays chaperone-like activity and takes part in proteasome-mediated proteins degradation; it is also involved in the control of cell cycle, growth and death and in the modulation of cell signaling. The eEF1A2 participates in the activation of kinases signaling pathways and in modulation of cytoskeletal organization by filipodia. Thus, eEF1A1 and eEF1A2 have similar canonical translation elongation functions, but they differ in the non-canonical functions, such as cytoskeleton modification [26,27] and protein degradation [28], as well as cell proliferation, migration and phosphatidylinositol signaling [29].

The role of eEF1A1 isoform in tumor onset and progression is less clear because of its ubiquitous expression; it is one of the most abundant protein in the cells. On the contrary, eEF1A2 was widely recognized to be an oncogene in many tumors.

For many human tumors, eEF1A2 is a putative oncogene because of its ectopic expression, that relates with tumor onset as firstly demonstrated in 2002, when Anand et al. found expression of eEF1A2 in 30% of ovarian tumors, but not in normal ovary cells [29]. Interestingly, it was known that a high proportion of ovarian and breast tumors had amplification of the 20q11 region, in which eEF1A2 maps, [30,31] and Anand et al., demonstrated that 14/53 of ovarian tumors brought amplifications of the region surrounding EEF1A2 gene [29]. In general, cancer development and aggressiveness of ovarian, breast, lung, gastric, hepatic and pancreatic cancers were associated with overexpression of eEF1A2 [32,33]. The eEF1A2 protein resulted to sustain both prostate cancer and hepatocarcinoma [34–37].

In breast cancers, the overexpression of eEF1A2 is related with a positive prognosis in two studies [38,39]. Moreover, in an analysis based on a transcription genes database, it resulted to be a negative factor for Distant Metastasis Free Survival in Luminal A while a positive factor for Post Progression Survival in HER2+ cancer subtypes [40]. However, the levels of eEF1A2 protein has not been dissected in the TNBC yet.

In this study, we evaluated, by an immunohistochemically fine-timing technique, the expression of eEF1A2 protein in formalin-fixed paraffin-embedded tissues of a cohort of TNBC patients to explore the potential significance of eEF1A2 as prognostic biomarker. For this purpose, we investigated the relationship between eEF1A2 levels and clinical-pathological status, clinical outcomes and some molecular information such as expression of p53, Ki-67, and androgen receptors (AR).

## Materials and methods

### Ethics statement

This study was a retrospective study. All the specimens were retrieved from the archive of pathological anatomy of the Azienda Sanitaria Universitaria Integrata di Trieste (ASUITS)-Cattinara Hospital. The study was approved by the Independent Ethics Committee (Comitato Etico Unico Regionale–CEUR) of Friuli Venezia Giulia and was conducted in accordance with the approved guidelines and regulations. All patients signed the informed consent and patients’ data were anonymized by assigning a numbered code. The Ethics Committee also remitted the informed consent.

### Study population

This retrospective single center cohort study was conducted on routinely recorded data extracted from the database of the Breast Unit of Trieste, Italy. The cohort included women with invasive TNBC surgically treated in ASUITS between January 2002 and December 2011. TNBC was defined as tumor with negative IHC for the estrogen (<1%), progesterone (<1%) and low or absent HER2-amplification (IHC zero or 1+ or negative in situ hybridization). Data on patients’ follow-up and causes of death were retrieved from the Computerized Medical Records of ASUITS.

Patients who had a previous history of breast cancer or distant metastasis at diagnosis were excluded. Women who underwent neoadjuvant chemotherapy or with no adjuvant chemotherapy treatment were also excluded to guarantee a study population as homogeneous as possible. Furthermore, women with unknown outcomes or with insufficient evaluable sample tissues were excluded.

Patient and tumor characteristics examined, have included age, type of surgery, histology, tumor size (pT), lymph node status (pN), stage, tumor grade, familiarity, adjuvant systemic treatment, and adjuvant radiotherapy.

Molecular information comprised the proliferation index Ki-67 (cut-off of positivity 20% [8], p53, androgen receptors (cut-off of positivity 10%) and eEF1A2 expression (obtained using IHC technique).

The main study end-points were Disease-Free Survival (DFS) and Breast Cancer-Specific Survival (BCSS). DFS was defined as the time from the date of surgery to the date of the first event, including local/regional disease recurrence, distant metastasis, invasive or in situ contralateral breast cancer, and second primary invasive cancer (non-breast cancer) according to recent guidelines [41]. BCSS was defined as the time from date of surgery to death from breast cancer. Deaths for other causes or patients lost to follow-up were censored. Last follow-up update on May 30, 2017.

### Immunohistochemistry procedure for eEF1A2 expression

Immunohistochemistry (IHC) was performed on formalin-fixed, paraffin-embedded tissue sections 3μm thin. Slides were placed for 20 minutes in 10mM sodium citrate buffer, pH 6.5 heat at 97°C in order to unmask antigens. To reduce nonspecific background staining due to endogenous peroxidase, specimens were incubated in UltraVision Hydrogen Peroxide Block (Thermo Scientific) for 10 minutes and after they were washed in Tris Buffer, for 5 minutes in Ultra Vision Protein Block (Thermo Scientific) to block nonspecific background staining. Sections were incubated for 60 minutes at room temperature with EF-1 alfa 2 (D-15 santa cruz-68481) to detect eEF1A2:sc-68481 mixed with EF-1 alfa1 (CBP-KK1): sc-21758 used at 1:300 dilution and after washed in Tris Buffer, for 30 minutes at room temperature with goat anti-rabbit IgG,F(ab’)2 –HRP: santa cruz-3837 used at 1:300 dilution. Finally, sections were incubated for 10 minutes with 3,3’ Diaminobenzidine chromogen (DAB Quanto–Thermo Scientific) and hematoxylin to nuclear counter stain.

As positive controls, we used staining intensity of eEF1A2 in normal breast parenchyma (external positive control) and staining intensity of eEF1A2 in normal breast parenchyma, if present in tumor tissue section (internal control).

### Interpretation of immunohistochemical staining

**Fig 1:**
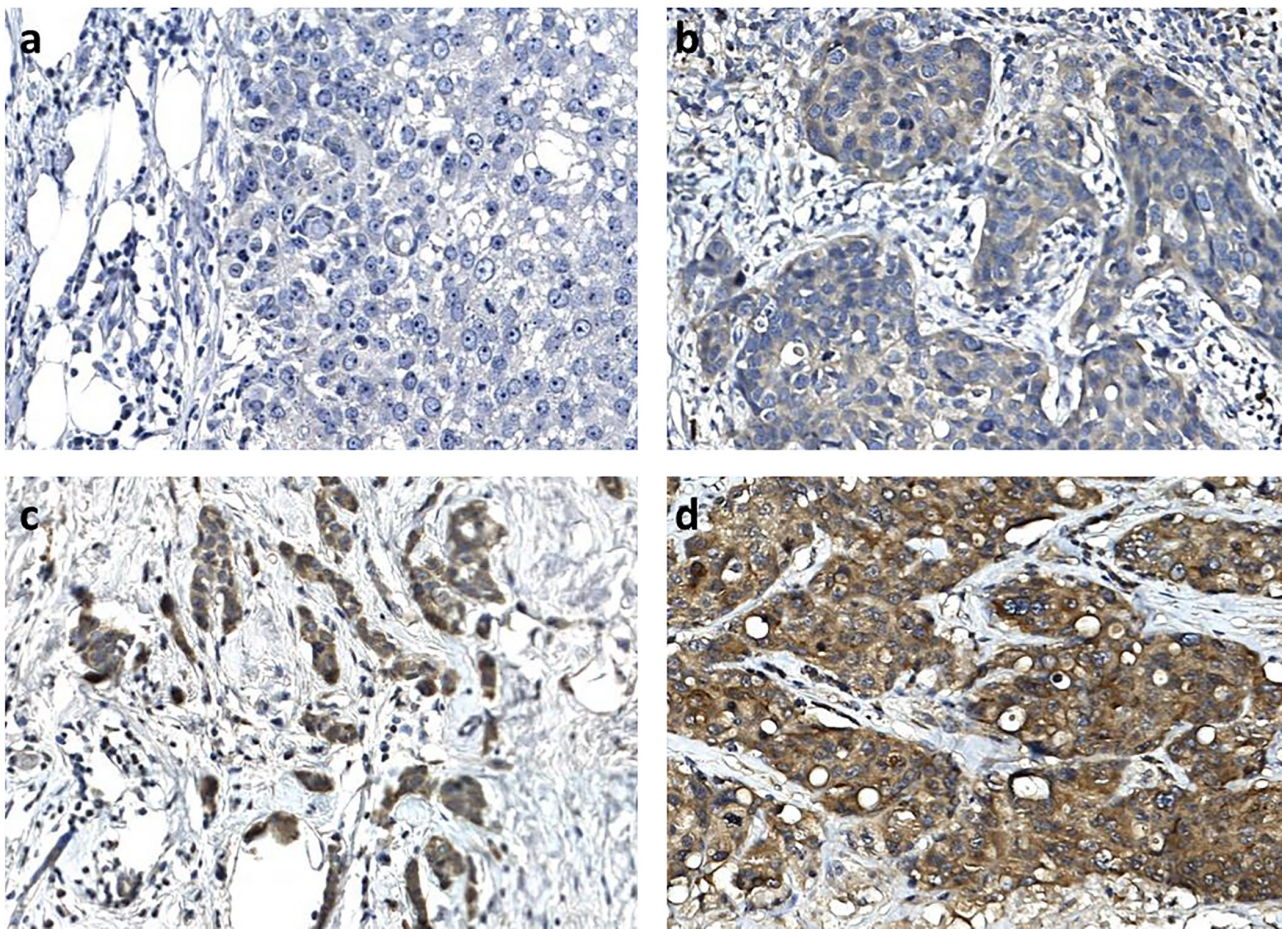
Immunohistochemical expression of eEF1A2 in breast cancer. a: absence of expression (0); b: weak expression (1+); c: moderate expression (2+); d: strong expression (3+). 20x magnification.

**Fig 2:**
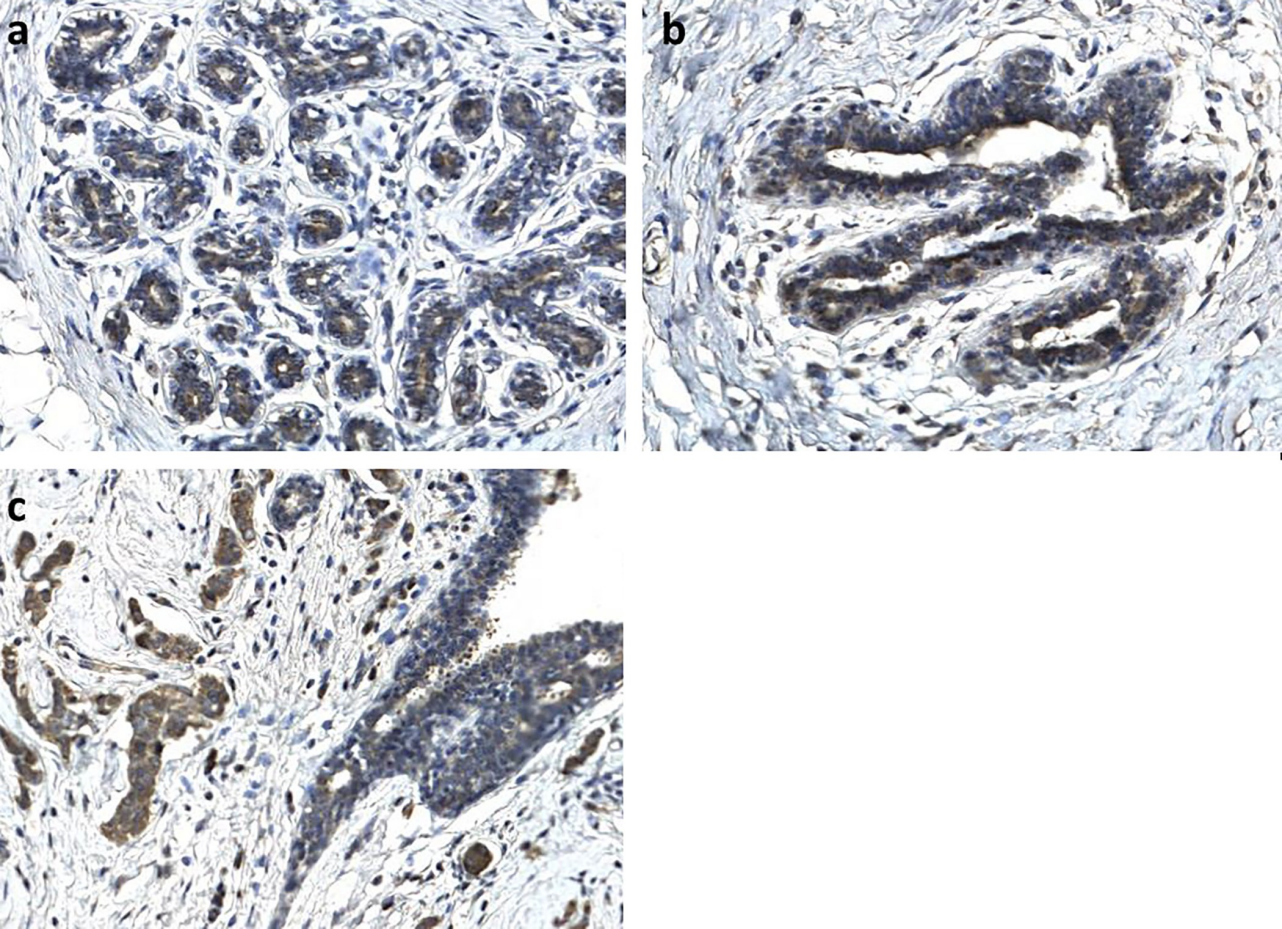
Expression of eEF1A2 in normal breast parenchyma. a: expression in acinar cells; b: expression in ductal cells; c: weak (1+) expression in normal ductal cells used to calibrate staining intensity in cancer cells (2+). 20x magnification.

The intensity of cytoplasmic staining for eEF1A2 was scored as 0 to 3+: 0, complete absence of staining in the cytoplasm and membrane of cancer cells or staining considerably weaker than normal breast acini; 1+, weak cytoplasmic staining or equal to normal breast acini; 2+, moderate cytoplasmic staining or slightly stronger than normal breast acini; 3+, strong cytoplasmic staining or markedly stronger than normal breast acini. The percentage of the expression of eEF1A2 was assigned by comparison with the positive internal controls. (see also Fig 1). eEF1A2 showed some expression in normal breast parenchyma. eEF1A2 expression was limited to the luminal side of epithelial ductal and acinar cells and was typically weak (1+) **(**Fig 2). Positivity in normal breast acini has been used to calibrate the assessment of positivity in neighboring cancer cells. Two pathologists evaluated each sample in a blinded manner. In case of discordance, the sample was revaluated or assigned to a third pathologist.

### Statistical analysis

We summarized data by using mean ± standard deviation (SD) or median and range, as appropriate, for continuous variables and absolute frequencies and percentages for categorical variables.

The eEF1A2 was studied by means of a semi-quantitative score but also as a dichotomous variable.

The eEF1A2 immunohistochemistry semi-quantitative score was calculated using the following formula: 0 × (percentage of cells staining absent [0]) + 1 × (percentage of cells staining weakly [1+]) + 2 × (percentage of cells staining moderately [2+]) + 3 × (percentage of cells staining strongly [3+]). In the literature, this score is called H-score and is often adopted to evaluate immunohistochemistry results of tumor samples [42,43].

Regarding dichotomization, different criteria were used. On the one side, we evaluated the effect of the eEF1A2 using cut-point values based on quantiles of the H score distribution such as, median and tertiles. On the other, we used two approaches less tied to data and not based on the H score distribution. The first considered “negative”, patients whose sum of the percentages of not-stained-cells [0] and weakly-stained-cells [1+] was greater than the sum of the percentages of moderately-stained-cells [2+] and strongly-stained-cells [3+]. The second considered “negative”, patients who had no expression of eEF1A2 (100% expression at 0 or 1+).

Inter-rater reliability of eEF1A2 measurements was assessed by different methods. When eEF1A2 was evaluated by means of the semi-quantitative H score were used both, the intraclass correlation coefficient (ICC) and the Bland Altman plot [44]. The ICC estimate and the 95% confidence intervals (CIs) were obtained using the “psych” R statistical package based on single rater, absolute-agreement, 2-way random effect model (ICC [2,1]) with 2 raters [45]. Even if any attempt to qualitatively define acceptable levels of reliability may be subject to criticism, the following guidelines were considered as general guidance for data interpretation. In particular, ICC values less than 0.5 were considered indicative of poor reliability, values between 0.5 and 0.75 indicative of moderate reliability, values between 0.75 and 0.9 indicative of good reliability, and values greater than 0.90 indicative of excellent reliability [45]. When eEF1A2 was evaluated by means of binary variables, inter-rater reliability was assessed by the Cohen’s Kappa coefficient. As mentioned previously, to aid in the interpretation of the kappa values, the classification proposed by Landis and Koch’s [46], was used: values equal or less than 0.20 were considered indicative of poor agreement, between0.21 and 0.40 of fair agreement, between 0.41 and 0.60 of moderate agreement, between 0.61–0.80 of good agreement, and between 0.81–1.00 of almost perfect agreement. However, the Cohen’s Kappa is sensitive to the unbalanced answers given by raters and to the overall prevalence of responses. For this reason, additional statistics were computed such as, the expected proportion of agreement, the proportion of positive agreement, the proportion of negative agreement, prevalence index (PI), the bias index (BI) and the prevalence adjusted bias kappa (PABAK). If PI and BI are equals to 0, means that there are almost no bias and prevalence effect on Cohen’s K. To compute these quantities the epiR package (function epi.kappa) of software R was used.

The association between H-score and clinical-pathological factors (age, type of surgery, pathologic tumor stage (pT), pathologic nodal stage (pN), grading (G), Ki67, p53, recurrences and death) were evaluated through Mann-Whitney test or Kruskall Wallis test, as appropriate.

The median follow-up was computed for censored patients, excluding women with the events of interest.

The study population was described with regards to the two time-to-event end-points (DFS and BCSS) by means of rates. These were computed as the ratio between the number of events and the sum of women-year at risk using STATA software and the “sptime” function. DFS and BCSS curves were plotted using the Kaplan-Meier method.

The association of the clinical-pathological and molecular factors with the two time-to-event end-points was analyzed separately using Cox proportional hazards regression. We checked proportional hazards assumption graphically and by means of the Therneau and Grambsch test [47]. The functional form of continuous or semi-continuous covariates such as, H score, was assessed by means of Martingale residuals. Hazard ratios (HRs) and their 95% CIs were reported. Statistically significant variables at 10% level at univariate analysis were selected as candidate prognostic factors for multivariate Cox analysis. Moreover, to choose the variables for the final multivariate model, it was important to consider the correlations among them, their clinical relevance as well as the concept of parsimonious modelling, because of the small number of events in this study. Since, in our study eEF1A2 had a large proportion of observations with an H score equals to zero, an additional analysis was performed in presence of a spike at zero (SAZ) covariate, according to the method proposed by Royston and Lorenz, [48–50]. Their method, called “FP-spike”, models the continuous non-zero observations of the SAZ covariate parametrically, using the fractional polynomials (FP), and adds to the Cox model a binary indicator variable (v), taking value equals to one when the SAZ covariate equals zero, and zero otherwise. The method consists of two stages: the first aims to identify the best FP function when also v is included in the model; the second to assess if v or the FP component can be eliminated without worsening the model fit. This second stage aims to understand if a sort of “dose-response” effect is present in the data and it is performed with the likelihood ratio test. As usual in Cox models, this method allows to consider the effect of other covariates into the model. Statistical analyses were conducted with R version 3.0.3 and STATA 14.2 (StataCorp, College Station, TX). All p-values (p) were two-tailed and, when not stated differently, differences were considered statistically significant when p < 0.05.

## Results

### Patient characteristics

**Fig 3:**
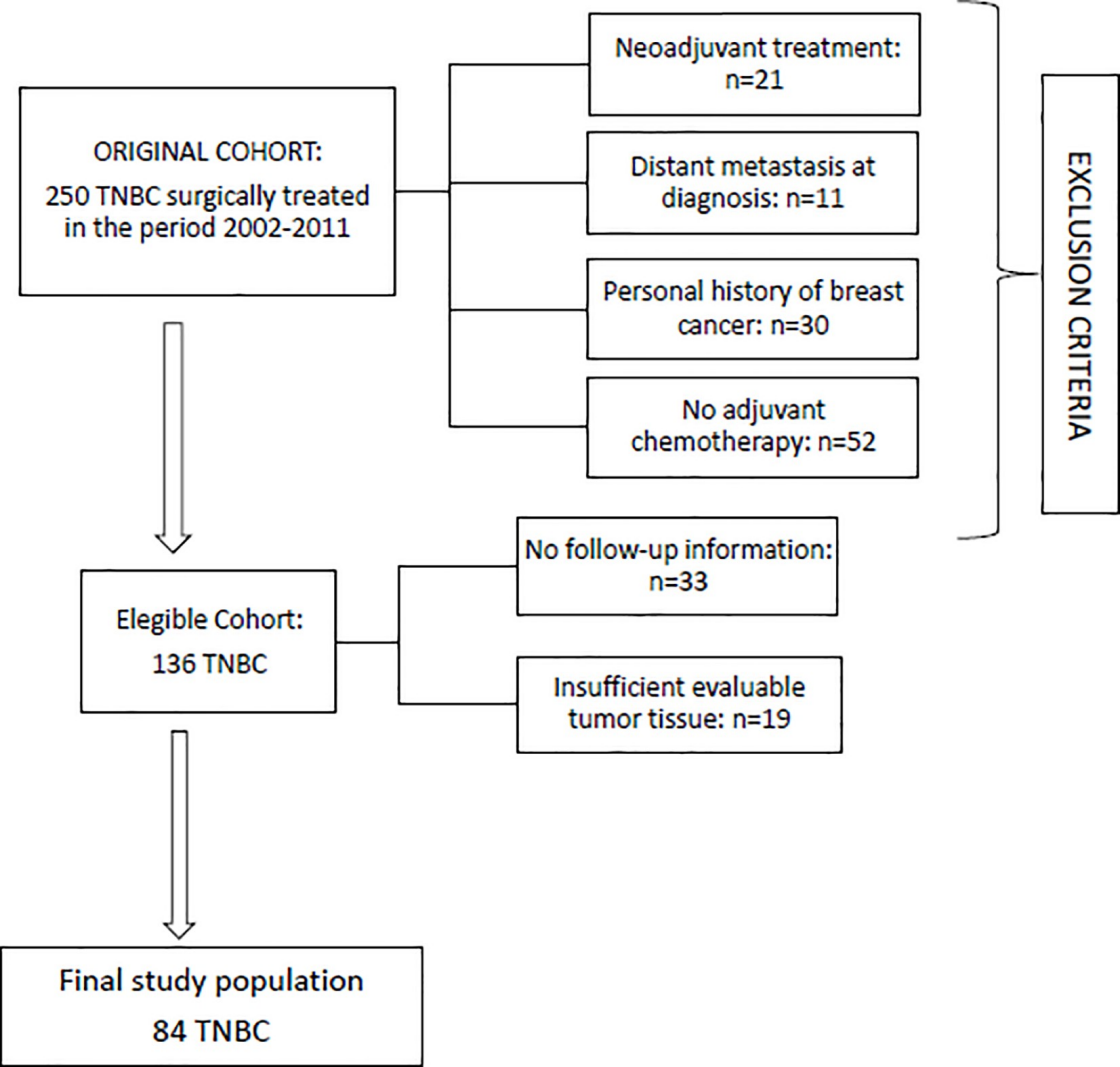
Flow-chart of the patient cohort included in this study.

The study enrolled a cohort of 250 TNBC women who received diagnosis of primary cancer from 2002 to 2011 and underwent to surgical treatment in ASUITS. We excluded 166 women (66.4%) for the following reasons: 21 received neoadjuvant treatment, 11 had distant metastases at diagnosis, 30 had personal history of breast cancer, 52 were no chemotherapy treated, 33 had no follow-up information and finally for 19 cases the tumor tissue was insufficient for the immunohistochemistry evaluation. Then, 84 women were eligible for the analysis (see Fig 3). The average age was 61 years old (range, 28–78 years), and advanced cancer (stage II or higher) was present in 44 patients (52.38%). 65 patients (77.38%) underwent conservative surgery while 19 patients (22.62%) mastectomy (Table 1**)**. These 19 women were candidate for mastectomy accordingly to current indications [51]; these types of cancer cannot be treated with conservative surgery for the following reasons: large tumor to breast size ratio (7), multicentric lesions (6), suspected genetic susceptibility subsequently proven (2), advanced (pT4) breast cancer (2), retroareolar carcinoma (1) and patient preference (1).

**Table 1:** Patients characteristics (n = 84).

Characteristics	n	%
**Age (years)**		
Median (min-max)	59 (28–78)	
**Type of Surgery**		
Conservative	65	77.38
Mastectomy	19	22.62
**Lymph nodal Surgery**		
Axillary Dissection	37	44.05
No Axillary Dissection	47	55.95
**Tumor Size**		
T1(<2 cm)	50	59.52
T2 (2–5 cm)	32	38.10
T3-4 (>5 cm)	2	2.38
**Invasive Cancers Histology Subtype**		
Ductal	48	57.14
Solide	17	20.24
Other (Medullary-Lobular- Apocrine)	19	22.62
**Lymph Node Metastasis**		
N0	54	64.29
N1mi	4	4.76
N+	26	30.95
**Stage**		
I	40	47.62
II	33	39.29
III	11	13.09
**Grade**^a^		
G1	1	1.28
G2	13	16.67
G3	64	82.05
**Ki67**		
<20%	15	17.86
> = 20%	69	82.14
**eEF1A2 (H-score)**		
Median (min-max)	10 (0–260)	
**eEF1A2**^b^		
Negative	77	92.77
Positive	6	7.23
**eEF1A2**^c^		
Negative	67	80.72
Positive	16	19.28
**AR**^a^		
Negative (<10%)	55	67.90
Positive (> = 10%)	26	32.10
**p53**		
Negative	28	33.33
Positive	56	66.67
**Family History of Breast Cancer**^a^		
No	49	62.03
First-degree relative	19	24.05
Second-degree relative	11	13.92
**Radiotherapy**^a^		
Yes	62	78.48
No	17	21.52
**Adjuvant Chemotherapy**		
Yes	84	100.00
No	0	0.00

^a^Numbers does not add up to the total due to missing values

^b^ Patient is considered eEF1A2 negative if the sum of the percentage of cells staining absent [0] and of the percentage of cells staining weakly [1+] is greater than the sum of the percentage of cells staining moderately [2+] and strongly [3+].

^c^ Patient was considered eEF1A2 negative if it had no expression of eEF1A2 (100% expression at 0 or 1+), and positive otherwise.

**Fig 4:**
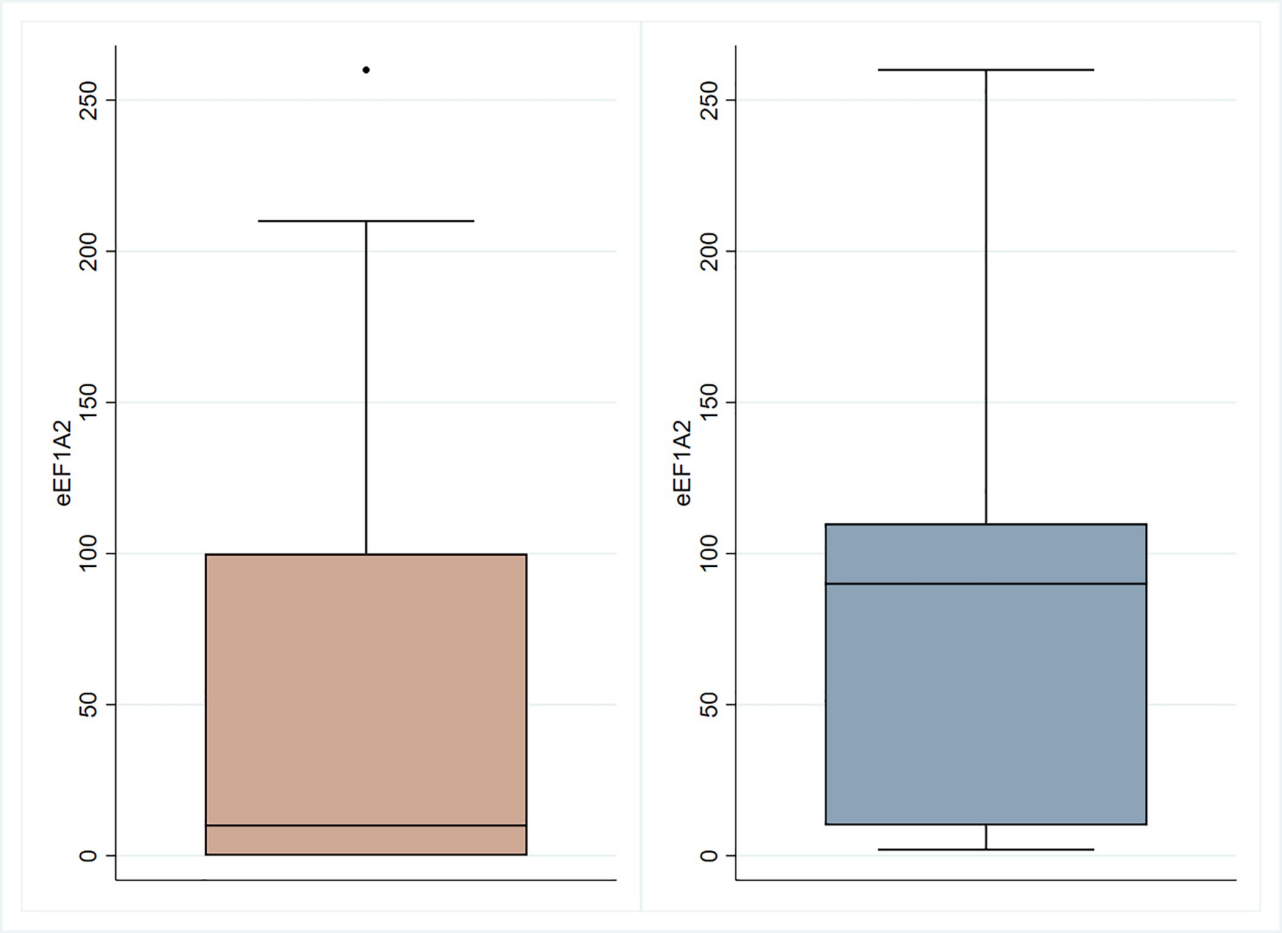
Box plots of eEF1A2 values according to presence (in the left) or exclusion (in the right) of 0 values.

Although ductal carcinoma NOS was the most frequent TNBC histologic subtype, a meaningful number of cases exhibited apocrine and medullary like features (9.52% and 5.95% respectively). The majority of the patients in our cohort had high Modified Bloom Richardson (MBR) pathologic grade (82.05%) and a significantly higher proportion of cases (82.14%) that were under high risk Ki67 category (> = 20%) as defined by St. Gallen international expert consensus recommendations. The median H-score value was 10 (range: 0–260), and tertiles respectively 0 and 56.69 (see Fig 4). Only 6 (7.23%) and 16 (19.28%) patients resulted eEF1A2 positive according to the dichotomization criteria reported in the statistical analysis section. High AR expression (≥10%) was found in 32.10% TNBC. A large percentage of patients (64.29%) had negative lymph nodes whereas 35.71% patients had metastatic lymph nodes. Dissection of the axillary lymph nodes was necessary in 37 patients (44.05%). All patients underwent post-operative adjuvant chemotherapy according to inclusion criteria.

During the follow-up period after surgery, relapse occurred in 31 patients (36.90%): 9 developed loco-regional recurrences and 21 distant metastases. The most common site of the first distant recurrence was the lung (47.62%, 10 out of 21 women with metastases). In 5 women, a contralateral breast cancer was diagnosed: 2 TNBC, 2 Luminal B Her 2 negative and 1 in situ breast cancer. Two patients had a second primary invasive cancer: an ovarian cancer and one choroid melanoma. 21 women died (25.00%) 18 of whom for breast cancer (after a disease relapse) and 3 for other causes.

### Analysis of eEF1A2 expression: Inter-rater reliability between two raters

Two pathologists independently assigned to each sample an immunohistochemical score in a blinded manner. A third senior pathologist revaluated the discrepant cases and discordance was adjudicated by consensus.

We conducted an inter-rater reliability analysis to evaluate consistency of eEF1A2 readings for H score by computing ICC and through Bland-Altman analysis (plot and statistics). The obtained ICC value was 0.72 (indicating moderate reliability) and its 95% confidence interval ranged between 0.60–0.80 (S1 Table). Scatter plot of measures of two raters and Bland-Altman Plot are shown in Supplementary material (S1 and S2 Figs): from Bland Altman plot we observed that most of measurements respected the Limit of Agreements and the points were scattered all over the place, above and below zero. Then it suggested that there was no consistent bias of one reading versus the other. Indeed, the value of mean bias of H-score was close to zero (0.94); it meant that on average, the second rater measured only 0.94 units more than the first one. (all Bland-Altman statistics are reported in S2 Table). We evaluated inter-rater reliability also for the two binary eEF1A2 variables: K statistics were respectively 0.64 and 0.52 (substantial agreement and moderate agreement). Since this statistic may be affected by bias and prevalence, S3 Table shows other useful statistics: for both binary variables, bias and prevalence were present, so the unadjusted kappa could not be considered a reliable indicator of measurements agreement and the PABAK should be preferred. This was equals to 0.88, substantial agreement, and 0.61, moderate agreement, respectively.

### Association between eEF1A2 expression and clinical-pathological parameters

Table 2 and S4 Table illustrate the association between eEF1A2 expression levels and clinical-pathological features, considered as a semi-continuous score or as a binary variable, respectively. eEF1A2 expression was higher in older women (p = 0.03) and associated with AR positive expression (p = 0.002). We did not observe other statistically significant association at 5% level.

**Table 2:** H score values in relation to baseline characteristics.

N (%)	H-Score	
Variable	Median (min-max)	p-value
**Age (Years)**		
<60	0 (0–210)	0.03
> = 60	17.50 (0–260)	
**Type of Surgery**		
Conservative	10 (0–260)	0.68
Mastectomy	5 (0–200)	
**Lymph nodal Surgery**		
Axillary Dissection	10 (0–205)	0.27
No Axillary Dissection	5 (0–260)	
**Tumor Size**		
T1(<2 cm)	5 (0–205)	0.82
T2 (2–5 cm)	10 (0–260)	
**Lymph Node Metastasis**		
N0	2.50 (0–205)	0.09
N+	15.00 (0–260)	
**Stage**		
I	7.50 (0–205)	0.76
II	7.50 (0–260)	
III	10 (0–210)	
**Grade**^a^		
G1-G2	50.30 (0–190)	0.30
G3	3.50 (0–260)	
**Ki67**		
<20%	80 (0–190)	
> = 20%	5 (0–260)	0.15
**p53**		
Negative	5 (0–210)	0.69
Positive	10 (0–260)	
**AR**^a^		
Negative (<10%)	0 (0–210)	0.002
Positive (> = 10%)	90 (0–260)	
**Family History of Breast Cancer**^a^		
No	5 (0–260)	0.63
First/Second -degree relative	10 (0–210)	
**Radiotherapy**^a^		
Yes	10 (0–260)	0.31
No	2 (0–140)	

^a^Numbers does not add up to the total due to missing values

### Analysis of DFS and BCSS with respect to standard prognostic factors

Median follow-up time was 9.05 years (5.55–15.35). Median DFS and BCSS were not reached: 5-year DFS and BCSS were 72.6%, 95% CI: 63.7%-82.8%; and 83.2%, 95% CI: 75.5%-91.6%, respectively. As shown in Table 3, conventional prognostic factors, including clinical tumor size, nodal status and disease stage were found to have a statistically significant association with DFS at univariate analysis (HR = 2.32, 95% CI: 1.14–4.71; HR = 2.64, 95% CI: 1.30–5.37; and HR = 8.90, 95% CI: 3.47–22.85, respectively). No statistically significant differences in DFS were observable between women with conservative surgery or mastectomy. Patient’s age was not associated with DFS too. We found similar results for BCSS, as illustrated in Table 4. Tumor size, node status and overall stage was found to be associated with an increased hazard of BCSS (hazard ratio respectively: HR = 3.72, 95% CI: 1.39–9.97; HR = 4.57, 95% CI: 1.70–12.24; and HR = 16.67, 95% CI: 4.81–57.72). Additionally, women aged over 60 years old, with mastectomy and axillary dissection, had poor prognosis (hazard ratio respectively: HR = 3.27, 95% CI: 1.07–9.95; HR = 3.16, 95% CI: 1.24–8.01; and HR = 2.57, 95% CI: 0.96–6.89).

**Table 3:** Results from univariate Cox models for DFS.

Characteristics	E	Women-years	Rates (x100)	HR (95% CI)	P-value
**Age (Years)**					
<60	9	167.51	5.37 (2.80–10.33)	1.00 (Reference)	0.44
> = 60	14	189.86	7.37 (4.38–12.45)	1.32 (0.64–2.71)	
**Type of Surgery**					
Conservative	15	290.55	5.16 (3.11–8.56)	1.00 (Reference)	0.20
Mastectomy	8	66.82	11.97 (5.99–23.94)	1.67 (0.76–3.63)	
**Lymph nodal Surgery**					
No Axillary Dissection	13	140.65	9.24 (5.37–15.92)	1.00 (Reference)	0.06
Axillary Dissection	10	216.72	4.61 (2.48–8.58)	1.97 (0.96–4.04)	
**Tumor Size**					
T1(<2 cm)	9	231.23	3.89 (2.03–7.48)	1.00 (Reference)	0.02
T2-3-4 (> = 2cm)	14	126.14	11.10 (6.57–18.74)	2.32 (1.14–4.71)	
**Lymph Node Metastasis**					
N0	10	249.26	4.01 (2.16–7.46)	1.00 (Reference)	0.008
N1mi-N1	13	108.11	12.02 (6.98–20.71)	2.64 (1.30–5.37)	
**Stage**					
I	6	188.37	3.19 (1.43–7.09)	1.00 (Reference)	
I	9	144.02	6.25 (3.25–12.01)	1.57 (0.67–3.64)	0.30
III	8	24.98	32.02 (16.01–64.03)	8.90 (3.47–22.85)	<0.001
**Grade**					
G1-G2	0	70.00	0	1.00 (Reference)	0.12
G3	22	260.88	8.43 (5.55–12.81)	2.32 (0.70–7.66)	
**Ki67**					
<20%	2	71.40	2.80 (0.70–11.20)	1.00 (Reference)	0.89
> = 20%	21	285.97	7.34 (4.79–11.26)	0.94 (0.38–2.30)	
**p53**					
Negative	7	119.10	5.88 (2.80–12.33)	1.00 (Reference)	0.28
Positive	16	238.27	6.72 (4.11–10.96)	1.60 (0.69–3.73)	
**AR**					
Negative (<10%)	13	234.84	5.54 (3.21–9.53)	1.00 (Reference)	0.18
Positive (> = 10%)	9	108.54	8.29 (4.31–15.94)	1.67 (0.79–3.53)	
**H score**^a^				1.04 (0.99–1.10)	0.11
**eEF1A2**^b^					
Negative	20	331.53	6.03 (3.89–9.35)	1.00 (Reference)	0.07
Positive	3	20.83	14.40 (4.64–44.64)	2.64 (0.91–7.61)	
**eEF1A2**^c^					
Negative	17	287.91	5.90 (3.67-9-50)	1.00 (Reference)	
Positive	6	64.46	9.31 (4.18–20.71)	1.53 (0.65–3.60)	0.33
**Family History of Breast Cancer**					
No	12	209.62	5.72 (3.25–10.08)	1.00 (Reference)	
First-degree relative	5	76.54	6.53 (2.72–15.70)	1.09 (0.45–2.63)	0.85
Second-degree relative	5	50.37	9.93 (4.13–23.85)	1.35 (0.50–3.68)	0.55
**Radiotherapy**					
No	13	277.43	4.69 (2.72–8.07)	1.00 (Reference)	0.16
Yes	5	69.40	7.20 (3.00–17.31)	0.54 (0.24–1.27)	

E = number of DFS events.

^a^ Results are reported as 5-unit increase.

^b^ Patient is considered eEF1A2 negative if the sum of the percentage of cells staining absent [0] and of the percentage of cells staining weakly [1+] is greater than the sum of the percentage of cells staining moderately [2+] and strongly [3+].

^c^ Patient was considered eEF1A2 negative if it had no expression of eEF1A2 (100% expression at 0 or 1+), and positive otherwise.

**Table 4:** Results from univariate Cox models for BCSS.

	E	Women-years	Rates (x100)	HR (95% CI)	P-value
**Age (Years)**					
<60	4	182.19	2.20 (0.82–5.85)	1.00 (Reference)	
> = 60	10	205.57	4.38 (2.28–8.41)	3.27 (1.07–9.95)	0.04
**Type of Surgery**					
Conservative	7	306.60	1.96 (0.88–4.36)	1.00 (Reference)	
Mastectomy	7	81.17	8.62 (4.11–18.09)	3.16 (1.24–8.01)	0.02
**Lymph nodal Surgery**					
No Axillary Dissection	10	162.79	6.14 (3.31–11.42)	1.00 (Reference)	
Axillary Dissection	4	224.98	1.33 (0.43–4.13)	2.57 (0.96–6.89)	0.06
**Tumor Size**					
T1(<2 cm)	3	239.91	1.25 (0.40–3.88)	1.00 (Reference)	
T2-3-4 (> = 2cm)	11	146.76	7.49 (4.15–13.53)	3.72 (1.39–9.97)	0.009
**Lymph Node Metastasis**					
N0	4	258.47	1.55 (0.58–4.12)	1.00 (Reference)	
N1mi-N1	10	128.21	7.80 (4.20–14.50)	4.57 (1.70–12.24)	0.003
**Stage**					
I	2	194.14	0.52 (0.07–3.66)	1.00 (Reference)	
II	5	157.95	3.17 (1.32–7.61)	1.94 (0.55–6.90)	0.3
III	7	35.67	19.63 (9.36–41.17)	16.67 (4.81–57.72)	<0.001
**Grade**					
G1-G2	0	70.00	0	1.00 (Reference)	
G3	14	288.90	4.85 (2.87–8.18)	-	
**Ki67**					
<20%	1	74.32	1.35 (0.19–9.55)	1.00 (Reference)	
> = 20%	13	313.44	3.83 (2.17–6.74)	1.19 (0.34–4.10)	0.79
**p53**					
Negative	4	128.97	3.10 (1.16–8.26)	1.00 (Reference)	
Positive	10	258.80	3.48 (1.81–6.68)	1.52 (0.60–4.66)	0.46
**AR**					
Negative (<10%)	10	253.31	3.95 (2.12–7.34)	1.00 (Reference)	
Positive (> = 10%)	4	118.37	3.38 (1.27–9.00)	0.88 (0.31–2.51)	0.82
**H score**^a^				1.05 (0.99–1.12)	0.08
**eEF1A2**^b^					
Negative	12	358.88	3.34 (2.00–5.89)	1.00 (Reference)	0.06
Positive	2	22.80	8.77 (2.19–35.08)	3.18 (0.92–10.99)	
**eEF1A2**^c^					
Negative	10	312.80	3.20 (1.72–5.94)	1.00 (Reference)	
Positive	4	68.88	5.81 (2.18–15.47)	2.48 (0.92–6.63)	0.07
**Family History of Breast Cancer**					
No	8	226.05	3.54 (1.77–7.08)	1.00 (Reference)	
First-degree relative	3	85.88	3.49 (1.13–10.83)	0.74 (0.20–2.71)	0.65
Second-degree relative	2	54.74	1.83 (0.26–12.97)	1.61 (0.50–5.14)	0.42
**Radiotherapy**					
No	6	294.18	2.04 (0.92–4.54)	1.00 (Reference)	
Yes	4	79.69	5.02 (1.88–13.37)	0.44 (0.14–1.34)	0.15

E = number of BCSS events.

^a^ Results are reported as 5-unit increase.

^b^ Patient is considered eEF1A2 negative if the sum of the percentage of cells staining absent [0] and of the percentage of cells staining weakly [1+] is greater than the sum of the percentage of cells staining moderately [2+] and strongly [3+].

^c^ Patient was considered eEF1A2 negative if it had no expression of eEF1A2 (100% expression at 0 or 1+), and positive otherwise

### Analysis of DFS and BCSS with respect to eEF1A2 expression

**Fig 5:**
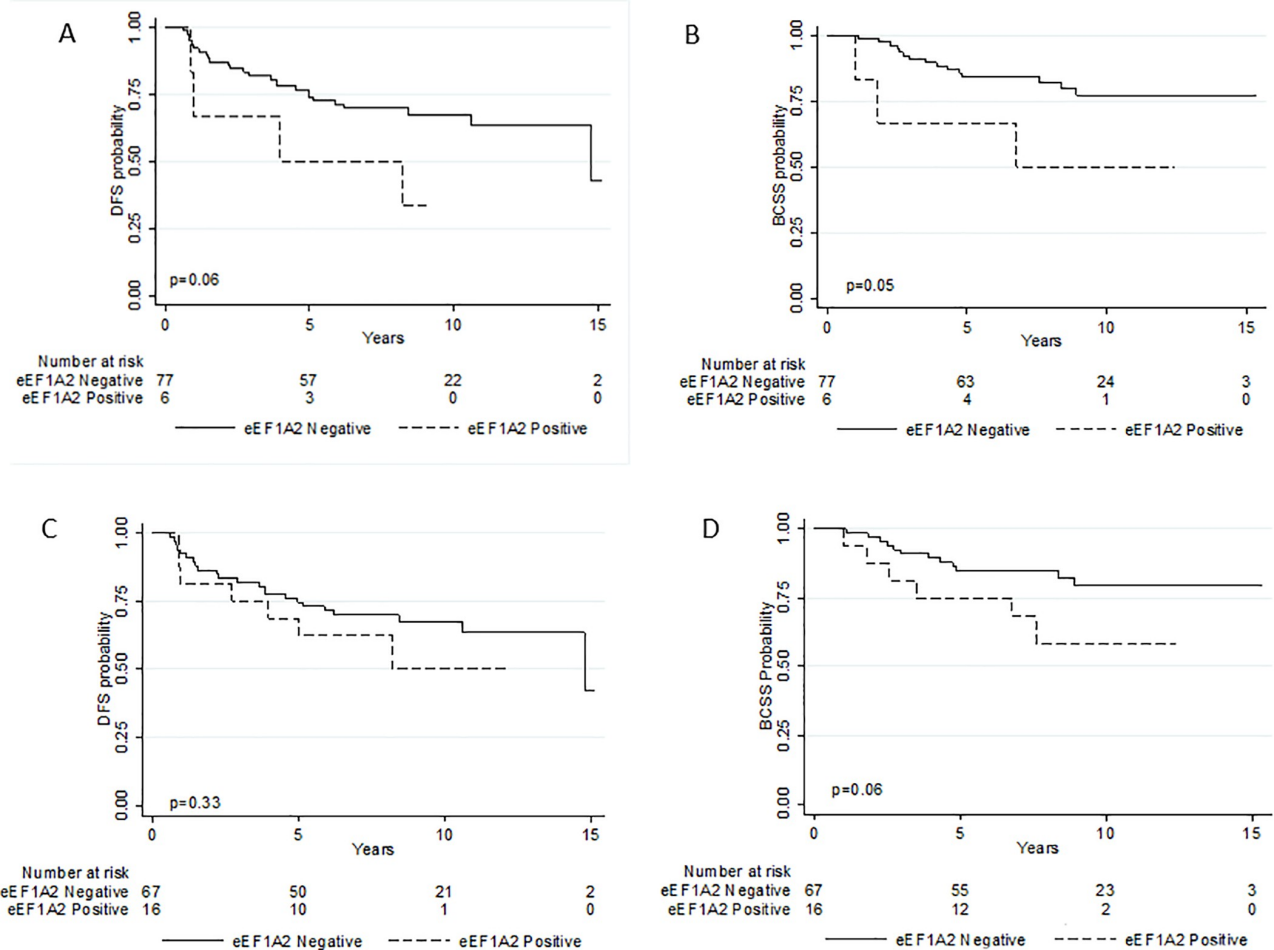
**Estimated disease-free survival (A-C) and breast cancer specific survival (B-D) according to eEF1A2 dichotomizations**. In panels A and B, a patient is considered eEF1A2 negative if the sum of the percentage of cells staining absent [0] and of the percentage of cells staining weakly [1+] is greater than the sum of the percentage of cells staining moderately [2+] and strongly [3+]; in panels C and D, a patient was considered eEF1A2 negative if it had no expression of eEF1A2 (100% expression at 0 or 1+), and positive otherwise.

At univariate analysis increasing values of eEF1A2 expression were associated with a worse prognosis even if results did not reach statistical significance at 5% level (HR = 1.04, 95% CI: 0.99–1.10, p = 0.11 for DFS and HR = 1.05, 95% CI: 0.99–1.12, p = 0.08 for BCSS), Tables 3 and 4. Considering the two dichotomous variables, results were in the same direction even if characterized by less precise estimates, Tables 3 and 4. Fig 5 shows the Kaplan-Meier curves for DFS and BCSS for the two dichotomous variables, respectively. The eEFIA2 H score dichotomized using the median value was not associated either with DFS or BCSS (p = 0.74 and p = 0.71 respectively). Analogous results were observed using tertiles as cut-off values for the H score, even if patients in the third tertile compared to those in the first one (with score value of zero), showed an increased hazard for the events of interest (p = 0.50 and p = 0.19 respectively).

Regarding DFS, adjusted for disease stage, higher values of the score were associated with a worst DFS (HR = 1.05, 95% CI: 1.007–1.105, p = 0.04), Table 5 –Model 1. Type of surgery, tumor size and lymph node status were correlated with tumor stage, but only the latter was evaluated for inclusion in the multivariable analysis. When dichotomous versions of eEF1A2 were considered, similar results were obtained. Compared to women with a dominance of cells with absent or weakly staining, women with more than 50%, of moderately or strongly stained cells as, had an HR = 2.61, 95% CI: 0.88–7.71, p = 0.08 (Table 5 –Model 2). Similar effects were observed also when a less strict definition of “positivity” was considered, HR = 1.80, CI: 0.75–4.33, p = 0.19, respectively (Table 5 –Model 3).

**Table 5:** Results from multivariate Cox models for DFS.

	*Model 1*		*Model 2*			*Model 3*		
	HR (95% CI)	P-value		HR (95% CI)	P-value		HR (95% CI)	P-value
**H score**^a^	1.05 (1.007–1.105)	0.04	**eEF1A2**^b^			**eEF1A2**^c^		
			Negative	1.00 (Reference)		Negative	1.00 (Reference)	
			Positive	2.61 (0.89–7.71)	0.08	Positive	1.80 (0.75–4.33)	0.19
**Stage**			**Stage**			**Stage**		
I	1.00 (Reference)		I	1.00 (Reference)		I	1.00 (Reference)	
II	1.41 (0.59–3.33)	0.44	II	1.46 (0.62–3.47)	0.37	II	1.42 (0.60–3.36)	0.43
III	9.80 (3.74–25.12)	<0.001	III	8.86 (3.40–23.03)	<0.001	III	9.34 (3.58–24.35)	<0.001

^a^ Results are reported as 5-unit increase.

^b^ Patient is considered eEF1A2 negative if the sum of the percentage of cells staining absent [0] and of the percentage of cells staining weakly [1+] is greater than the sum of the percentage of cells staining moderately [2+] and strongly [3+].

^c^ Patient was considered eEF1A2 negative if it had no expression of eEF1A2 (100% expression at 0 or 1+), and positive otherwise

Regarding BCCS, disease stage and eEF1A2 expressed by the H score, showed again independent prognostic roles, Table 6 –Model 1. Higher values of the score were associated with worst prognosis (HR = 1.07, 95% CI: 1.01–1.14, P-value = 0.026). Similar results were observed for the two dichotomous variables (HR = 4.25, 95% CI: 1.16–15.54, P-value = 0.029 and HR = 2.95, CI: 1.07–8.09, p = 0.036, respectively), Table 6 –Models **2** and 3.

**Table 6:** Results from multivariate Cox models for BCSS.

	*Model 1*		*Model 2*			*Model 3*		
	HR (95% CI)	P-value		HR (95% CI)	P-value		HR (95% CI)	P-value
**H score**^a^	1.07 (1.008–1.140)	0.026	**eEF1A2**^b^			**eEF1A2**^c^		
			Negative	1.00 (Reference)		Negative	1.00 (Reference)	
			Positive	4.25 (1.16–15.55)	0.029	Positive	2.95 (1.07–8.09)	0.036
**Stage**			**Stage**			**Stage**		
I	1.00 (Reference)		I	1.00 (Reference)		I	1.00 (Reference)	
II	1.80 (0.51–6.43)	0.36	II	1.91 (0.54–6.77)	0.23	II	1.83 (0.51–6.51)	0.32
III	17.44 (5.02–60.63)	<0.001	III	16.57 (4.78–57.40)	<0.001	III	16.13 (4.70–55.37)	<0.001

^a^ Results are reported as 5-unit increase.

^b^ Patient is considered eEF1A2 negative if the sum of the percentage of cells staining absent [0] and of the percentage of cells staining weakly [1+] is greater than the sum of the percentage of cells staining moderately [2+] and strongly [3+].

^c^ Patient was considered eEF1A2 negative if it had no expression of eEF1A2 (100% expression at 0 or 1+), and positive otherwise.

For DFS, a simple linear function (FP1) to model the relationship between the non-zero values of the H score and the hazard was selected by the FP-spike procedure, Table 7. Moreover, when the indicator variable, *v*, and FP1 component were tested for removal, both resulted important for the model fit (P-values = 0.032 and P-value = 0.006, respectively). As already shown, adjusting for stage, we observed that increasing values of eEF1A2 were associated with poor prognosis. The FP spike model showed that also women with an H score of zero, have a risk of a recurrence higher compared to women with values different from zero (P-value = 0.039). For BCSS, by the FP-spike analysis (Table 7), the addition of the binary component did not bring any substantial contribution to the model fit. In particular, the best functional relationship between eEF1A2 and the hazard of was described by a linear model confirming that an increase of H-score values was associated with an increase in worse prognosis.

**Table 7:** Results from multivariate Cox models for DFS and BCSS using the FP-Spike approach for eEF1A2.

	DFS		BCSS	
	log HR (95% CI)	P-value	log HR (95% CI)	P-value
*First stage*^a^				
***v***	1.07 (0.05–2.09)	0.039	0.41 (-0.87–1.68)	0.53
**FP1: Linear**	0.009 (0.003–0.156)	0.003	0.008 (0.0007–0.0162)	0.032
**Stage**				
I	(reference)		(reference)	
II	0.40 (-0.47–1.27)	0.364	0.60 (-0.67–1.88)	0.353
III	2.44 (1.45–3.42)	<0.001	2.86 (1.62–4.10)	<0.001
*Second stage*^b^				
FP1 (dropping *v*)		0.032		0.531
*v* (dropping FP1)		0.006		0.040

Log HR, logarithm of the hazard ratio; FP1, first degree fractional polynomial.

^a^ In the first stage of PF-spike procedure, the full model including both the indicator *v*, eEF1A2 and other covariates is fitted. The selected model is taken then taken to the second stage.

^b^ In the second stage both *v* and eEF1A2 are tested for removal.

## Discussion

TNBC is a heterogeneous disease, highly variable with respect to its biology and etiology and more likely to be poorly differentiated; these cancers often display an aggressive clinical course [52]. Moreover, due to the lack of specific cellular receptors in cancer cells, targeted therapies have not been established, and, as a result, TNBC mortality remains high [11,52]. Many studies documented a high rate of recurrence among TNBC patients [53,54]. In our TNBC cohort we found a distant recurrence rate of 36.90%, in agreement with literature data [53]. The risk of distant recurrences appeared to be high in the first 1–4 years after diagnosis and treatment [54,55]: our findings confirmed this, as more than 70% of the recurrences occurred within 4 years, with a peak during the 1 year. In our TNBC cohort, on univariate analysis, tumor size, nodal status and stage were associated with DFS and BCSS, in agreement with a recent study [56,57]. Moreover, we found that women with a conservative surgery exhibited an improved BCSS in comparison to patients treated with mastectomy (univariate analysis) as recently shown by other authors [14,57].

The eEF1A2 protein delivers aminoacyl tRNAs to the ribosome and it is selectively expressed in specialized tissues such as heart, skeletal muscles and brain [58]. Several studies demonstrated that eEF1A2 acts as a growth-enhancing protein in many human cancers. Its ectopic expression has been shown to favor oncogenesis by stimulating the phospholipid signaling pathway and the Akt-dependent cell migration [59].

There is also evidence that eEF1A2 overexpression is predictive of patient’s prognosis in various epithelial cancers, which are sometimes positive and sometimes negative [38,40,60,61]. Recently, other studies have reported that the upregulation of eEF1A2 predicted a prolonged survival in ovarian [62] and in HER2 negative breast cancer [38]. The difference in prognostic significance of eEF1A2 overexpression might be due to the different mechanisms that are implicated in and on the pathways that are altered in tumors [63]. Two studies reported that the overexpression of eEF1A2 in tumors did not completely depend on the genomic status of this locus, suggesting instead that it depends on an increase of the transcription and/or on mRNA stability [29,34]. The regulatory mechanisms of eEF1A2 overexpression in tumors is complex and it can involve miRNA too [64].

In this study, we determined eEF1A2 protein expression levels by IHC in TNBC tissue and assessed its prognostic role with respect to DFS and BCSS. To our knowledge, this is the first study investigating eEF1A2 protein expression in tissue samples of TNBC. Different statistical approaches were considered to evaluate the association with clinical outcomes. Firstly, we summarized eEF1A2 expression by means of the semi-quantitative H score and, after the adjustment for tumor stage, a statistically significant association between increasing values of the biomarker and the increase of hazards for adverse events was observed (recurrence-DFS or death-BCSS). Similar results were obtained for BCSS with other statistical methods (FP-spike model) taking the high proportion of zeros in the H score into account [48–50]. However, for DFS the FP-spike model gave a statistically significant adverse effect in women with an H score of zero compared to those with a positive one. The interpretation of this observation deserves further investigations, maybe by evaluating eEF1A1 isoform levels. In fact, it has been demonstrated a reciprocal influence of the two proteins at expression levels in many cancers and the possibility that the overexpression of eEF1A1 protein sustains cancer progression [40,65]. The use of categorical variables based on quantiles of the H score distribution (median and tertiles) was marginally treated in this paper due to the limits of the H score itself and to the main data dependence on such measures.

Differently from our evidence, another study found that the overexpression of eEF1A2 protein was a predictor of good prognosis in breast cancer [38]. In this regard, eEF1A2 expression in other tumor entities showed differing results: some studies attributed a higher expression of eEF1A2 with a poor prognosis (pancreatic ductal adenocarcinoma [60] and localized prostate cancer [66]), while others found that it was associated with a favorable outcome (non-small cell lung cancer [67] and breast cancer [38]). It is worth to underline that with respect to 80% of pancreatic cancers, only 30%-60% of breast cancer showed overexpression of eEF1A2 [38], underlying the heterogeneity of eEF1A2 expression in breast cancers. Our evidence suggests that in TNBC high levels of eEF1A2 protein sustain cancer aggressiveness. Moreover, most of the studies analyzing eEF1A2 [33,38,66], quantified mRNA expression whereas in our study we measured protein levels that might give different results [65]. An explanation may reside in the fact that mRNA quantity is often not quantitatively equivalent to protein levels. In particular, the half-lives of eEF1A2 protein is of about 95 hours, whereas the half-lives of the mRNAs are shorter (about 60 hours) [68]. It is conceivable that high levels of eEF1A2 protein led to a worse prognosis in TNBC by sustaining cell survival, migration and invasion, by the ability of the protein to upregulate MMP-9, to activate PKR and to suppress PI3K/Akt/NF-kB signaling pathway [61, 69,70].

Notably, in our TNBC cohort, eEF1A2 expression was higher in older women (p = 0.03) and was associated with AR positive expression (p = 0.002). We did not observe any statistically significant association between eEF1A2 expression and type of surgery, tumor size or stage, Ki-67, p53, grading and family history of breast cancer in accordance with a large cohort study of 438 primary breast cancer specimen [38]. We obtained similar results considering a different definition of DFS that is, excluding contralateral breast cancers and second primary invasive cancers (S5 Table).

Further studies using a larger independent data set of TNBCs are necessary to confirm the prognostic value of eEF1A2 protein before an implementation in clinical practice. Certainly, the measurement of protein level is very interesting because immunohistochemistry is the main technique available in most pathology labs to evaluate a marker. It is worth noting that from inter-rater reliability analysis, considering the calculated measures of reliability, H-score resulted reproducible (ICC = 0.72 95% CI = 0.60–0.80), also by using the two categorized scores (PABAK = 0.61 and 0.88). In our cohort, we are aware of the fact that the use of the H score and of categorical variables derives from its distribution, it may assign sometimes the same score or the same class to women with quite different patterns, making the interpretation difficult. This is a limit of IHC due to the heterogeneity of the tumors among the tissues (i.e. different percentage of positive cells and different cell staining intensity). The evaluation of mRNA expression levels in formalin-fixed, paraffin-embedded tissue could contribute to clarify the potency of eEF1A2 as prognostic biomarker in TNBC, as well as to assign a score in doubt cases. In this respect, it is outstanding the agreement of our evidence on eEF1A2 protein levels with the annotation of eEF1A2 mRNA expression levels in TNBC in Kaplan Meier Plotter [71]. In particular, we chose gene expression array database obtained from JetSet best probe set analysis to assure an unbiased quality score for probe set, and from patients with systemic treatments. In those patients with high expression levels of eEF1A2 mRNA a shorter DFS was recorded (25 months) with respect to the DFS of the low expression cohort (47.51 months) with a p value = 0.024; HR = 1.64 (1.06–2.54).

## Conclusion

In conclusion, our data showed that high expression levels of eEF1A2 protein in formalin-fixed paraffin-embedded TNBC tissues is associated with worse prognosis. These results encourage extending the study on eEF1A2 expression levels in a larger independent cohort of TNBC to evaluate its prognostic usefulness for the clinical practice and possibly as potential target for the therapy.

## Supporting information

S1 Fig Scatter plot Rater 1 vs Rater 2. (TIF)

S2 Fig Bland Altman plot (H-score). (TIF)

S1 Table ICC estimates for continuous score. (DOCX)

S2 Table Bland-Altman Statistics. (DOCX)

S3 Table Kappa Statistics and other concordance indices. (DOCX)

S4 Table eEF1A2 expression in relation to baseline characteristics. (DOCX)

S5 Table Factors influencing disease-free survival (DFS) -Univariate analysis. (DOCX)

S6 Table Raw data: Clinic-pathologic features, survival and eEF1A2 data of TNBC cohort. (XLSX)
