# Correction: Elevated levels of eEF1A2 protein expression in triple negative breast cancer relate with poor prognosis

**DOI:** 10.1371/journal.pone.0227068

**Published:** 2019-12-19

**Authors:** Fabiola Giudici, Elisabetta Petracci, Oriana Nanni, Cristina Bottin, Maurizio Pinamonti, Fabrizio Zanconati, Bruna Scaggiante

Affiliation 2 for authors Elisabetta Petracci and Oriana Nanni is incomplete. The correct affiliation 2 is: Unit of Biostatistics and Clinical Trials, Istituto Scientifico Romagnolo per lo Studio e la Cura dei Tumori (IRST) IRCCS, Meldola, Italy.
