# Supplementary figures and images for: Elevated levels of eEF1A2 protein expression in triple negative breast cancer relate with poor prognosis
Source: PLoS One. 2019 Jun 20;14(6):e0218030. doi: 10.1371/journal.pone.0218030 (PMC6586289; doi:10.1371/journal.pone.0218030)

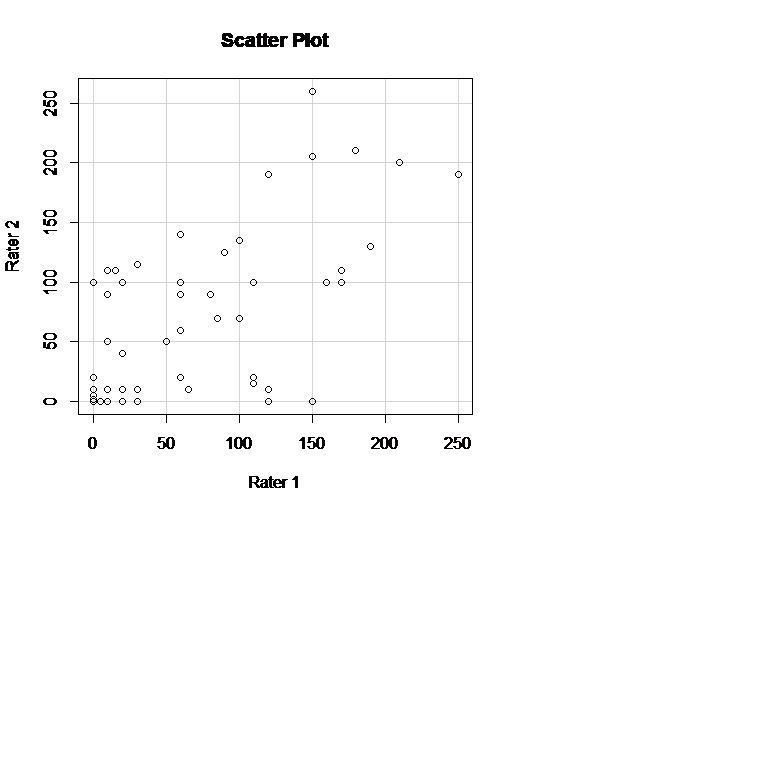

Supplement: S1 Fig — (TIF) [file pone.0218030.s001.tif]

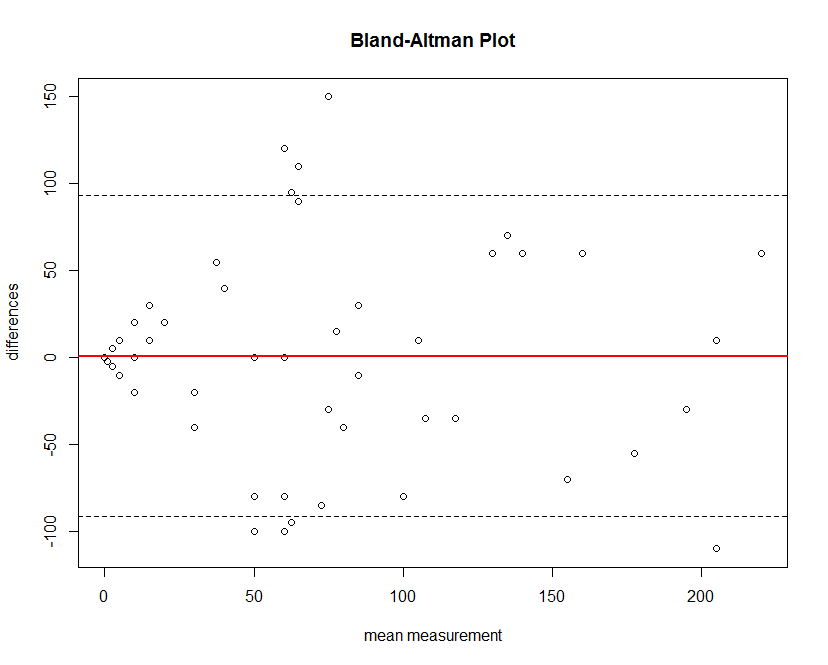

Supplement: S2 Fig — (TIF) [file pone.0218030.s002.tif]
