# Supplementary material for: Elevated levels of eEF1A2 protein expression in triple negative breast cancer relate with poor prognosis
Source: PLoS One. 2019 Jun 20;14(6):e0218030. doi: 10.1371/journal.pone.0218030 (PMC6586289; doi:10.1371/journal.pone.0218030)
Supplement: S1 Table — (DOCX) [file pone.0218030.s003.docx]

**S1 Table** ICC estimates for continuous score

|  |  | **95% Confidence Interval** | | **F test with True Value 0** | | |
| --- | --- | --- | --- | --- | --- | --- |
| **Score** | **ICC** | **Lower Bound** | **Upper Bound** | **Value** | **(df1,df2)** | **p-value** |
| **H-score** | 0.72 | 0.597 | 0.801 | 6.07 | (82,82) | <0.001 |
