# Supplementary material for: Elevated levels of eEF1A2 protein expression in triple negative breast cancer relate with poor prognosis
Source: PLoS One. 2019 Jun 20;14(6):e0218030. doi: 10.1371/journal.pone.0218030 (PMC6586289; doi:10.1371/journal.pone.0218030)
Supplement: S2 Table — (DOCX) [file pone.0218030.s004.docx]

**S2 Table** Bland-Altman Statistics

| **Indicators** | **H-score** |
| --- | --- |
| **Mean bias±1.96 SD** | 0.94±92.37 |
| **95% Confidence Interval of Mean Bias** | (-9.35;11.23) |
| **Limits of Agreement (LOA)** | (-91.43; 93.31) |
| **95% Confidence Interval of lower LOA** | (-109.25;-73.61) |
| **95% Confidence Interval of upper LOA** | (75.49;111.14) |
