# Supplementary material for: Elevated levels of eEF1A2 protein expression in triple negative breast cancer relate with poor prognosis
Source: PLoS One. 2019 Jun 20;14(6):e0218030. doi: 10.1371/journal.pone.0218030 (PMC6586289; doi:10.1371/journal.pone.0218030)
Supplement: S3 Table — (DOCX) [file pone.0218030.s005.docx]

**S3 Table** Cohen’s Kappa and other statistics

| **Indicators** | **eEF1A2**^a^ | **eEF1A2^b^** |
| --- | --- | --- |
| ***K coefficient*** | 0.64, 95% CI: 0.43-0.84 | 0.52, 95% CI: 0.32 0.72 |
| ***P_e_*** | 0.83 | 0.60 |
| ***P_pos_*** | 0.67 | 0.64 |
| ***P_neg_*** | 0.97 | 0.87 |
| ***PI*** | 0.82 | 0.47 |
| ***BI*** | 0.04 | 0.14 |
| ***PABAK*** | 0.88,95% CI: 0.73-0.96 | 0.61, 95% CI: 0.41-0.77 |

^a^ Patient is considered eEF1A2 negative if the sum of the percentage of cells staining absent (0) and of the percentage of cells staining weakly (1+) is greater than the sum of the percentage of cells staining moderately (2+) and strongly (3+).

^b^Patient was considered eEF1A2 negative if it had no expression of eEF1A2 (100% expression at 0 or 1+), and positive otherwise.

P_e_=expected proportion of agreement; P_pos_= proportion of positive agreement; P_neg_=proportion of negative agreement; PI=prevalence index, BI=bias index; PABAK= prevalence-adjusted and bias-adjusted kappa;
