# Supplementary material for: Elevated levels of eEF1A2 protein expression in triple negative breast cancer relate with poor prognosis
Source: PLoS One. 2019 Jun 20;14(6):e0218030. doi: 10.1371/journal.pone.0218030 (PMC6586289; doi:10.1371/journal.pone.0218030)
Supplement: S4 Table — (DOCX) [file pone.0218030.s006.docx]

**S4 Table** eEF1A2 expression in relation to baseline characteristics.

|  | **eEF1A2^b^** | | | **eEF1A2^c^** | | |
| --- | --- | --- | --- | --- | --- | --- |
| **Characteristics** | **Negative (n = 77)**  **n (%)** | **Positive (n = 6)**  **n (%)** | **p-value** | **Negative (n = 67)**  **n (%)** | **Positive (n=16) n (%)** | **p-value** |
| **Age (Years)** |  |  |  |  |  |  |
| <60 | 38 (49.35) | 1 (16.67) | 0.12 | 35(52.24) | 4 (25.00) | 0.05 |
| >=60 | 39 (50.65) | 5 (83.33) |  | 32 (47.76) | 12 (75.00) |  |
| **Type of Surgery** |  |  |  |  |  |  |
| Conservative | 59 (76.63) | 5 (83.33) | 0.71 | 52 (77.61) | 12 (75.00) | 0.99 |
| Mastectomy | 18 (23.34) | 1 (16.67) |  | 15 (22.39) | 4 (25.00) |  |
| **Lymph nodal Surgery** |  |  |  |  |  |  |
| Axillary Dissection | 32 (41.56) | 4 (66.67) | 0.23 | 27 (40.30) | 9 (56.25) | 0.25 |
| No Axillary Dissection | 45 (58.44) | 2 (33.33) |  | 40 (59.70) | 7 (43.75) |  |
| **Tumor Size** |  |  |  |  |  |  |
| T1(<2 cm) | 46 (59.74) | 3 (50.00) | 0.64 | 40 (59.70) | 9 (56.26) | 0.80 |
| T2 (2-5 cm) | 31 (40.26) | 3 (50.00) |  | 27 (40.30) | 7 (43.75) |  |
| **Lymph Node Metastasis** |  |  |  |  |  |  |
| N0 | 52 (67.53) | 2 (33.33) | 0.09 | 46 (68.66) | 8 (50.00) | 0.16 |
| N+ | 25 (32.47) | 4 (66.67) |  | 21 (31.34) | 8 (50.00) |  |
| **Stage** |  |  |  |  |  |  |
| I | 38 (49.35) | 2 (33.33) | 0.34 | 33 (49.25 ) | 7 (43.75) | 0.76 |
| II | 30 (38.96) | 2(33.33) |  | 26 (38.81) | 6 (37.50) |  |
| III | 9 (11.69) | 2 (33.33) |  | 8 (11.94) | 3 (18.75) |  |
| **Grade^a^** |  |  |  |  |  |  |
| G1-G2 | 12 (16.90) | 1 (16.67) | 0.78 | 12 (18.46) | 1 (8.33) | 0.68 |
| G3 | 59 (83.10) | 5 (83.33) |  | 53 (81.54) | 11 (91.67) |  |
| **Ki67** |  |  |  |  |  |  |
| <20% | 13 (16.88) | 1 (16.67) | 0.99 | 11 (16.42) | 3 (18.75) | 0.99 |
| >=20% | 64 (83.12) | 5 (83.33) |  | 56 (83.58) | 13 (81.25) |  |
| **p53** |  |  |  |  |  |  |
| Negative | 27 (35.06) | 1 (16.67) | 0.66 | 23 (34.33) | 5 (31.25) | 0.82 |
| Positive | 50 (64.94) | 5 (83.33) |  | 44 (65.66) | 11 (68.75) |  |
| **AR^a^** |  |  |  |  |  |  |
| Negative (<10%) | 53 (69.74) | 2 (40.00) | 0.05 | 50 (74.64) | 5 (35.71) | 0.009 |
| Positive (>=10%) | 23 (30.26) | 3 (60.00) |  | 17 (25.37) | 9 (64.29) |  |
| **Family History of Breast Cancer^a^** |  |  |  |  |  |  |
| No | 46 (63.89) | 3 (50.00) | 0.50 | 40 (64.52) | 9 (56.25) | 0.57 |
| First/Second -degree  relative | 26 (36.11) | 3 (50.00) |  | 22 (35.48) | 7 (43.75) |  |
| **Radiotherapy^a^** |  |  |  |  |  |  |
| Yes | 58 (77.33) | 3 (100) | 0.38 | 50 (76.92) | 11 (84.61) | 0.72 |
| No | 17 (22.67) | 0 (100) |  | 15 23.08) | 2 (15.39) |  |

^a^Numbers does not add up to the total due to missing values. ^b^ Patient is considered eEF1A2 negative if the sum of the percentage of cells staining absent (0) and of the percentage of cells staining weakly (1+) is greater than the sum of the percentage of cells staining moderately (2+) and strongly (3+).

^c^ Patient was considered eEF1A2 negative if it had no expression of eEF1A2 (100% expression at 0 or 1+), and positive otherwise.
