# Supplementary material for: Elevated levels of eEF1A2 protein expression in triple negative breast cancer relate with poor prognosis
Source: PLoS One. 2019 Jun 20;14(6):e0218030. doi: 10.1371/journal.pone.0218030 (PMC6586289; doi:10.1371/journal.pone.0218030)
Supplement: S5 Table — (DOCX) [file pone.0218030.s007.docx]

**S5 Table** Factors influencing disease-free survival (DFS) -Univariate analysis

| **Variable** | **UNIVARIATE COX ANALYSIS (1)** | | **UNIVARIATE COX ANALYSIS (2)** | |
| --- | --- | --- | --- | --- |
|  | **HR (95% CI)** | **p-value** | **HR (95% CI)** | **p-value** |
| **Age (Years)** |  |  |  |  |
| <60 | 1.00 (Reference) |  | 1.00 (Reference) |  |
| >=60 | 1.32 (0.64-2.71) | 0.44 | 2.18 (0.91-5.27) | 0.07 |
| **Type of Surgery** |  |  |  |  |
| Conservative | 1.00 (Reference) |  | 1.00 (Reference) |  |
| Mastectomy | 1.67 (0.76-3.63) | 0.2 | 2.55 (1.11-5.83) | 0.03 |
| **Lymph nodal Surgery** |  |  |  |  |
| No Axillary Dissection | 1.00 (Reference) |  | 1.00 (Reference) |  |
| Axillary Dissection | 1.97 (0.96-4.04) | 0.06 | 2.98 (1.27-6.97) | 0.01 |
| **Tumor Size** |  |  |  |  |
| T1(<2 cm) | 1.00 (Reference) |  | 1.00 (Reference) |  |
| T2-3-4 (>=2cm) | 2.32 (1.14 -4.71) | 0.02 | 2.64 (1.17-5.95) | 0.01 |
| **Lymph Node Metastasis** |  |  |  |  |
| N0 | 1.00 (Reference) |  | 1.00 (Reference) |  |
| N1mi-N1 | 2.64 (1.30-5.37) | 0.008 | 3.98 (1.74-9.12) | 0.001 |
| **Stage** |  |  |  |  |
| I | 1.00 (Reference) |  | 1.00 (Reference) |  |
| II | 1.57 (0.67-3.64) | 0.3 | 2.01 (0.72-5.65) | 0.19 |
| III | 8.90 (3.47-22.85) | <0.001 | 13.46 (4.63-39.09) | <0.001 |
| **Grade*** |  |  |  |  |
| G1-G2 | 1.00 (Reference) |  | 1.00 (Reference) |  |
| G3 | 2.32 (0.70-7.66) | 0.12 | 2.98(0.70-12-70) | 0.14 |
| **Ki67** |  |  |  |  |
| <20% | 1.00 (Reference) |  | 1.00 (Reference) |  |
| >=20% | 0.94 (0.38-2.30) | 0.89 | 0.91 (0.34-0.86) | 0.86 |
| **p53** |  |  |  |  |
| Negative | 1.00 (Reference) |  | 1.00 (Reference) |  |
| Positive | 1.60 (0.69-3.73) | 0.28 | 1.89 (0.72-5.08) | 0.21 |
| **AR*** |  |  |  |  |
| Negative (<10%) | 1.00 (Reference) |  | 1.00 (Reference) |  |
| Positive (>=10%) | 1.67 (0.79-3.53) | 0.18 | 1.03 (0.42-2.53) | 0.94 |
| **Family History of Breast Cancer*** |  |  |  |  |
| No | 1.00 (Reference) |  | 1.00 (Reference) |  |
| First-degree relative | 1.09 (0.45-2.63) | 0.85 | 1.36 (0.51-3.61) | 0.54 |
| Second-degree relative | 1.35 (0.50-3.68) | 0.55 | 1.46 (0.47-4.54) | 0.51 |
| **Radiotherapy*** |  |  |  |  |
| No | 1.00 (Reference) |  | 1.00 (Reference) |  |
| Yes | 0.54 (0.24-1.27) | 0.16 | 0.43 (0.17-1.10) | 0.08 |
| *missing |  |  |  |  |

1) Events: loco-regional recurrences, distant metastasis, contralateral breast cancer and second primary invasive cancer (non-breast cancer)

2) Events: loco-regional recurrences, distant metastasis
